# NMR-based metabolic characterization of chicken tissues and biofluids: a model for avian research

**DOI:** 10.1007/s11306-016-1105-7

**Published:** 2016-09-15

**Authors:** Caroline Ivanne Le Roy, Luke John Mappley, Roberto Marcello La Ragione, Martin John Woodward, Sandrine Paule Claus

**Affiliations:** 1 Department of Food and Nutritional Sciences, University of Reading, Whiteknights, Reading, RG6 6AP UK; 2 Department of cancer research, University College London, London, UK; 3 Faculty of Health and Medical Sciences, School of Veterinary Medicine, University of Surrey, Guilford, Surrey, GU2 7AL UK

**Keywords:** Chicken, Metabolome, Nuclear magnetic resonance spectroscopy (NMR), Metabolite

## Abstract

**Introduction:**

Poultry is one of the most consumed meat in the world and its related industry is always looking for ways to improve animal welfare and productivity. It is therefore essential to understand the metabolic response of the chicken to new feed formulas, various supplements, infections and treatments.

**Objectives:**

As a basis for future research investigating the impact of diet and infections on chicken’s metabolism, we established a high-resolution proton nuclear magnetic resonance (NMR)-based metabolic atlas of the healthy chicken (*Gallus gallus*).

**Methods:**

Metabolic extractions were performed prior to ^1^H-NMR and 2D NMR spectra acquisition on twelve biological matrices: liver, kidney, spleen, plasma, egg yolk and white, colon, caecum, faecal water, ileum, pectoral muscle and brain of 6 chickens. Metabolic profiles were then exhaustively characterized.

**Results:**

Nearly 80 metabolites were identified. A cross-comparison of these matrices was performed to determine metabolic variations between and within each section and highlighted that only eight core metabolites were systematically found in every matrice.

**Conclusion:**

This work constitutes a database for future NMR-based metabolomic investigations in relation to avian production and health.

**Electronic supplementary material:**

The online version of this article (doi:10.1007/s11306-016-1105-7) contains supplementary material, which is available to authorized users.

## Introduction

The Food and Agriculture Organization of the United Nation (FAOSTAT: http://www.fao.org/home/en/), calculated that approximately 22 billion chickens were produced commercially worldwide in 2012, China being the main producer with over 5 billion birds. A major production issue in commercial systems is animal density that is favourable for rapid spread of disease. Most chicks receive a cocktail of vaccines at hatch or even *in ovo*, but remain susceptible to typical production related endemic disease and other food borne zoonosis such as *Salmonella* or *Campylobacter* (Boer and Hahné 1990; Dufrenne et al. 2001). All infections represent a large potential economic loss for the chicken industry and is one of the main cause of meat contamination by food born pathogens (Tessari et al. 2009; White et al. 1997). Vaccines and antibiotics are commonly used to tackle such infections in order to stop spread and symptoms and minimize the associated cost. With regard to antibiotic use, increasing antimicrobial resistance has been observed in animal farming and has become a major concern in recent decades, stimulating the development of alternative treatments (McEwen and Fedorka-Cray 2002; Casewell et al. 2003). Therefore, in the interest of improving animal welfare and product quality, new more specific treatments are needed. Finally in the same purpose, attention is brought towards improving animal feeding. Chichen feed generally consists of a mix of grounded grains (corn, rice, wheat) and proteins most often from soya beans. However, the grain/protein ratio is different for egg laying and meat production. There are numerous added supplements including certain amino acids, minerals and oils. In addition feed is supplemented with vitamins A, D3 and riboflavine and mineral salts.

Multi-‘omics’ approaches help to gain better understanding of host-pathogen-drug interactions (Nicholson et al. 2004; McDermott et al. 2011). This consists in using together genomic (study of the genome) (Klug et al. 2012), transcriptomic (study of gene expression) (Bernot 2004), proteomic (studying the proteome) (Blackstock and Weir 1999) and metabonomic (studying the metabolome). Chicken genomic (Burta et al. 1995), transcriptomic (Murphy 2009) and proteomic (Doherty et al. 2004; Mann 2007; Mann and Mann 2008) data have already been published but, to date, none of them have reported a detailed analysis of the chicken metabolome. Metabonomic has been mainly developed for clinical and nutritional (nutrimetabonomics) research (Nicholson et al. 2002; Holmes et al. 2011; Solanky et al. 2003; Claus and Swann 2013) and allows to look at quantitative and qualitative metabolic variations caused by genetic mutation or environmental stress in a sample set (Nicholson and Wilson 2003). The nutrimetabonomics approach is therefore useful to evaluate the impact of nutrition and food on the host systemic metabolism and understand the dietary impact on productivity in livestock farming.

This paper presents the annotated NMR metabolic profiles of twelve chicken biological matrices to serve as reference for future studies. We selected four major biological matrices for the host systemic metabolism: liver, kidney, spleen and plasma. In addition, samples from the digestive system, including: colon, caecum, ileum and faecal water were analysed. Three relevant to industrial production and that could be used to evaluate or assess product quality: egg (yolk and white) and pectoral muscle. Finally brain cortex was also analysed.

## Materials and methods

### Animal husbandry and sample collection

Five 15–16 weeks of age NovoGen Brown commercial laying hens (*Gallus gallus*) were purchased from the Animal and Plant Health Agency (APHA) in Surrey. Animal husbandry conformed to animal Home Office licence (PPL 70/7249) and all procedures were performed in compliance with the Animals Scientific Procedures Act, 1986. Animals were provided with food (Chicken Layers Pellets, Dodson & Horrell—Composition detail in Material supplement) and water ad libitum. After 1 week of acclimatization (see food composition in supplement), animals of 15 weeks of age and weighing on average 1000 g (n = 6) were sacrificed by cervical dislocation. Tissues were sampled aseptically immediately after euthanasia and snap frozen in liquid nitrogen (−195.79 °C) and then transferred at −80 °C for storage until analysis. The following tissues were sampled: liver (right lobe), the right kidney, half longitudinal cut of the spleen, the right lobe of the prefrontal cortex, the middle of the external surface of the left pectoral muscle. Digestive tract samples were washed with PBS before freezing and faeces were collected directly by emptying the colon. One cm of proximal colon was sampled and 2 cm of the end on the left caecum were taken, 2 cm of ileum were sampled approximately 3 cm before the caecum. Plasma was sampled by *post*-*mortem* cardiac puncture. Egg yolk and white (n = 6) were sampled from randomly chosen eggs laid by older animals that had just come into lay (18 week old) from the same cohort of birds on the same diet and kept within the same environment.

### Sample preparation

Sample biopsies were homogenised using a bead beater (Qiagen, TissueLyser LT) at a frequency of 1/25 for 10 min for the digestive tract tissue and the muscle and 3 min for the liver, the spleen, the kidney and the cortex using glass beads. For this step, 0.1 g of tissue were homogenised in 1 mL of a 3:1 (v/v) MeOH/H_2_O solution for polar metabolite extraction. After centrifugation 10 min at 12 000×*g*, 0.9 mL of supernatant was dried in speed vacuum for 4.5 h at 45 °C and resuspended in 600 μL of phosphate buffer (pH 7.4) 0.2 M containing 90 % of D_2_O and 10 % of H_2_O plus 0.01 % of sodium 3-(tri-methylsilyl)-propionate-2,2,3,3-d_4_ (TSP) for NMR reference. Samples were then transferred into 5 mm NMR tubes for analysis. Egg yolk and white were prepared following the same protocol. Plasma samples were mixed at a 2:1 (v/v) ratio with phosphate saline buffer with 90 % D_2_O, of which, 500 µL were then transferred into 5 mm NMR tubes. Faecal samples were extracted by mixing 0.1 g of faeces in 1 mL of phosphate buffer (plus TSP) with a bead beater for 3 min using glass beads at the frequency of 1/25. Samples were centrifuged at 12 000×*g* for 10 min in a refrigerated centrifuge and supernatants were kept at 4 °C overnight to let urea precipitate. After centrifugation for 5 min at 12,000×*g*, the supernatant was transferred into 5 mm NMR tubes.

### NMR spectra acquisition

For all polar tissue extracts, egg yolk and faeces, ^1^H-NMR spectra were acquired on a Bruker Advance DRX spectrometer operating at 700.19 MHz and equipped with a CryoProbe™ from the same manufacturer. A standard 1-dimensional noesypr1D pulse sequence (noesypr1d 90° pulse length of 7.7 µs and total acquisition time 3.34 s) with water presaturation applied during relaxation delay (2 s) and a mixing time of 100 ms at 298 K was used. Plasma and egg white ^1^H NMR spectra were acquired using a Carr-Purcell-Meiboom-Gill (CPMG) (Meiboom and Gill 1958) pulse sequence to limit signal contribution from albumin and ovalbumin respectively. CPMG were acquired with simple presaturation of the water peak and a total spin–spin relaxation delay (2nτ) of 120 ms was used with the following sequence (90°-ts-180°-ts-FID). For each sample 256 scans (16 dummy scans) were recorded into 64 K data points over a spectral width of 12019 Hz as for noesypr1D. ^1^H–^1^H COSY and ^1^H–^13^C HSQC were obtained for each biological matrix on one representative sample for metabolite identification purposes.

### Data processing and analysis

Prior to Fourier transformation, an exponential window with line broadening of 0.3 Hz was applied to each 1D NMR spectrum. All spectra were phased manually and baseline corrected on MestReNova software (2013 Mestrelab Research S.L.). Spectral calibration was performed using TSP (δ 0.00) for all tissues and yolk samples, lactate (δ 1.33) for plasma and the H1 proton of α-glucose (δ 5.23) for egg white spectra. One representative spectrum was selected from each biological matrix for illustration purpose and peak assignments. Each peak was associated to a metabolite in accordance to available database such as HMDB or previously published papers. If a molecule presented a signature with several peaks, the presence of all the peaks for this same compound was assessed prior to validation by 2D NMR experiment such as COSY and HSQC. For these spectra signal suppression was done at δ 4.84 during FID processing using a MestReNova function (with the convolution option) to attenuate water resonance.

Signal assignment and metabolite identification was done using an in house standard database, published literature (Merrifield et al. 2011; Claus et al. 2008; Nicholson et al. 1995) and online public databases: the human metabolome data base (HMDB, http://www.hmdb.ca) and the magnetic resonance data bank (BMRB, http://www.bmrb.wisc.edu).

### Statistical analysis

For statistical analysis, spectra were imported into MatLab (version R2013b, The MathsWorks inc.) and residual signal water region was removed (δ 4.70–5.10) before normalisation (to account for variations in sample size and distribution) using a median-base probabilistic quotient method (Dieterle et al. 2006). Principal component analysis (PCA) was performed using algorithms provided by the Korrigan toolbox (Korrigan Sciences Ltd) in order to evaluate dominant sources of variation between biological matrices. Venn diagrams were also created using online Venny software (Venny 2.1 http://bioinfogp.cnb.csic.es/tools/venny/).

## Results and discussion

Systemic Metabolic characterisation of several mammals, including rodents (Claus et al. 2008; Griffin et al. 2000; Martin et al. 2007; Martin et al. 2009a, b), pig (Merrifield et al. 2011), humans (Ndagijimana et al. 2009; Holmes et al. 1997; Nicholson et al. 1995) and horse (Escalona et al. 2014) is available but, to date, no overview of any bird metabolic phenotype has been published despite their industrial significance and worldwide source of protein. This work gives a summary of the metabolic composition of twelve biological matrices detectable by NMR spectroscopy in order to be used for future NMR-based metabonomics research.

**Fig. 1:**
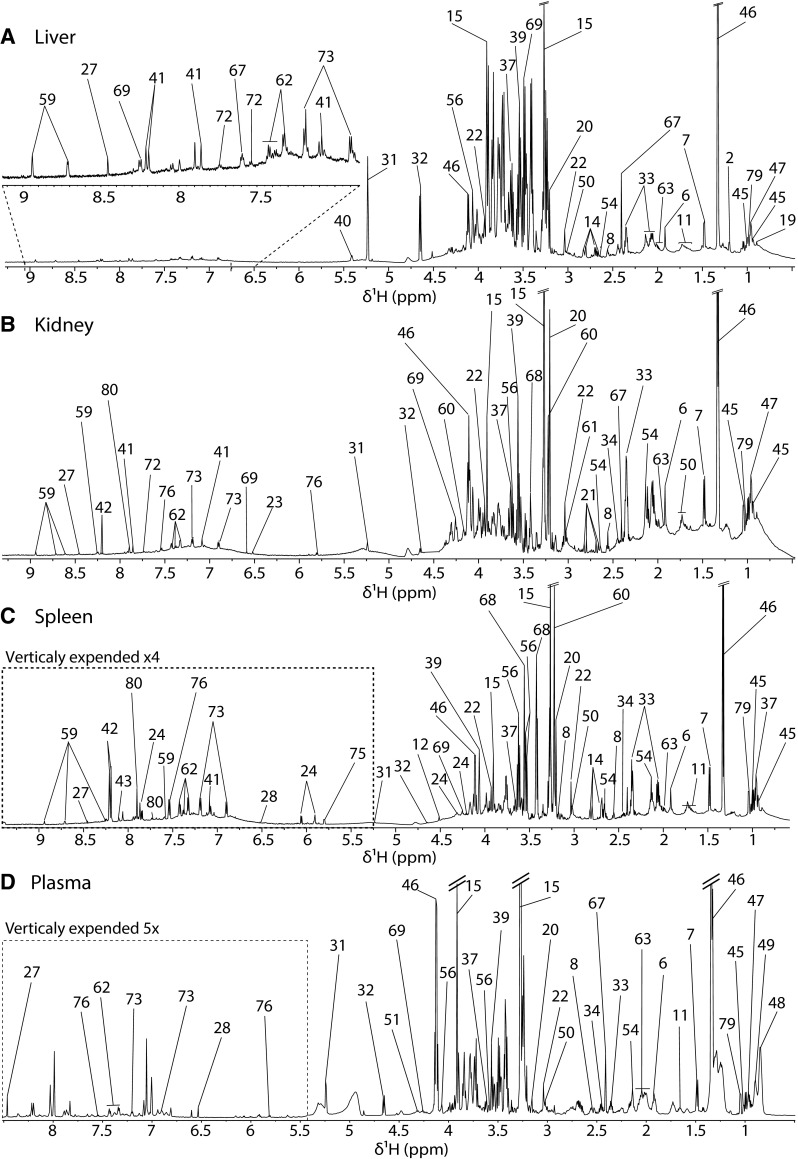
Partially assigned 700 MHz 1D NMR spectra of chicken liver, kidney, spleen and plasma. Numerical key described in Table 1

**Fig. 2:**
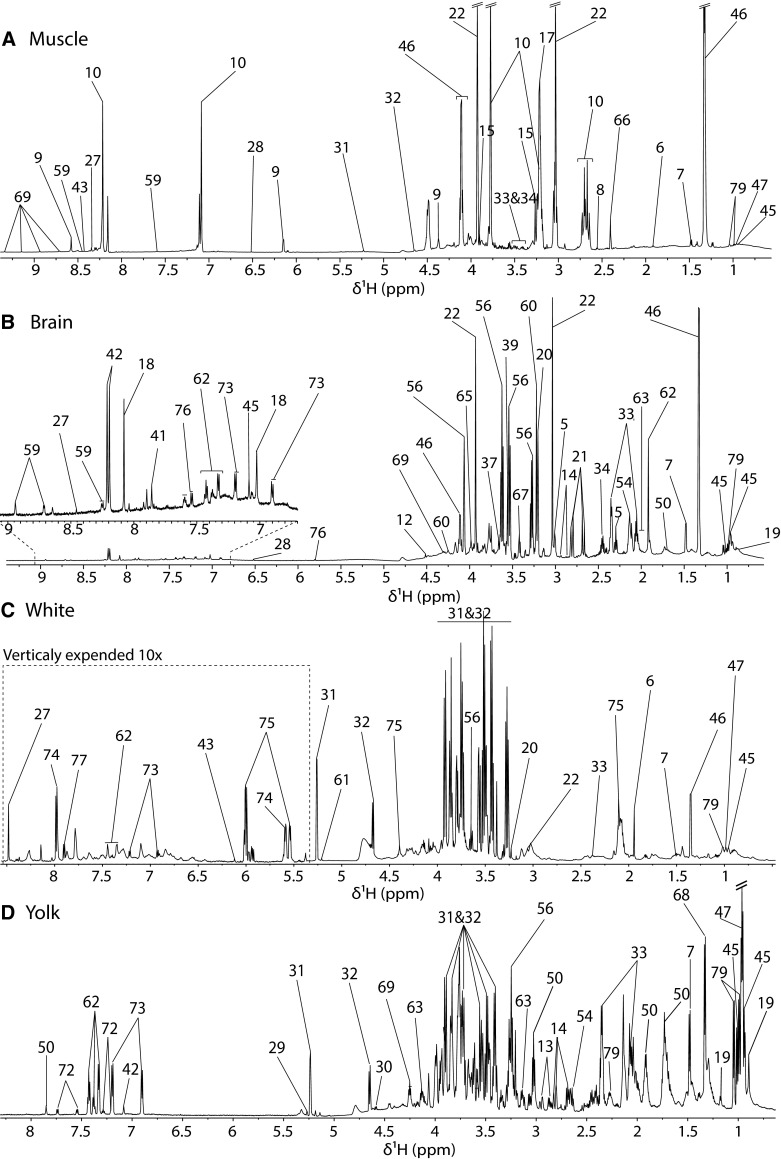
Partially assigned 700 MHz 1D NMR spectra of chicken muscle, egg white and yolk and cortex. Numerical key described in Table 1. In the figure, write egg white and egg yolk

**Fig. 3:**
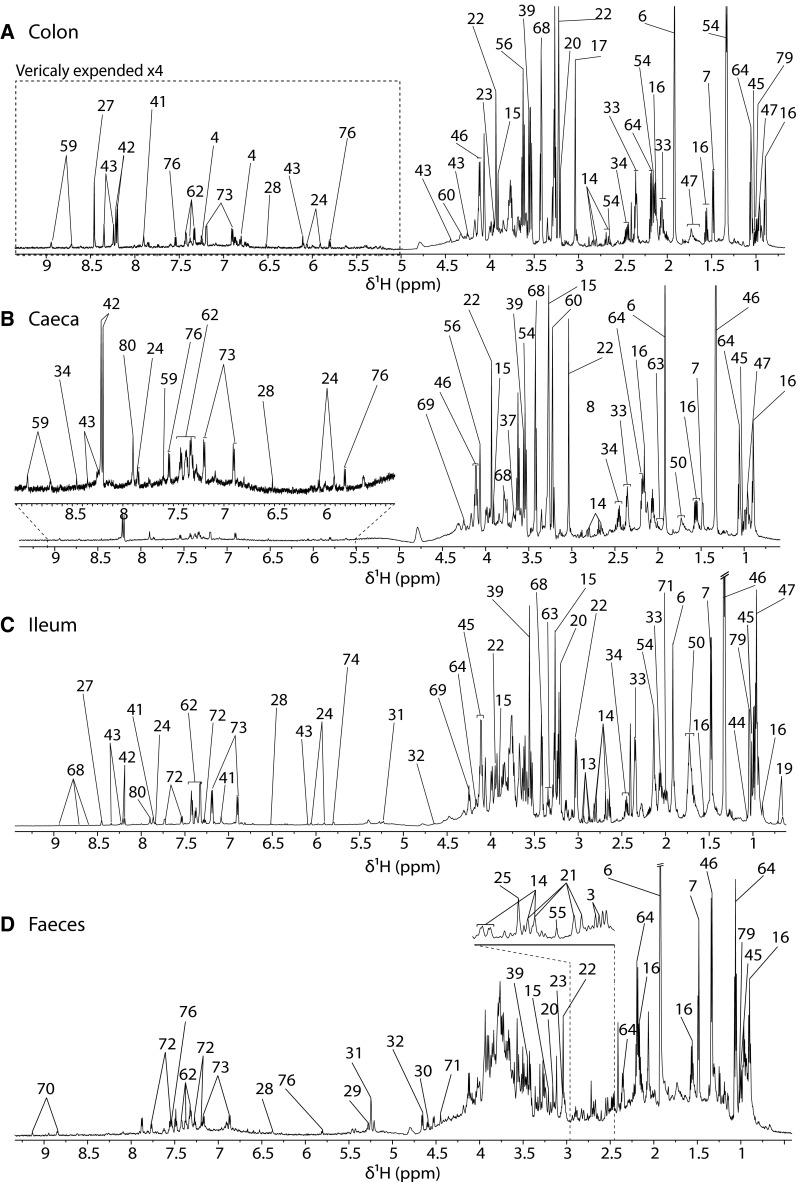
Partially assigned 700 MHz 1D NMR spectra of chicken colon, caecum, ileum and faeces. The Numerical key is described in Table 1

**Fig. 4:**
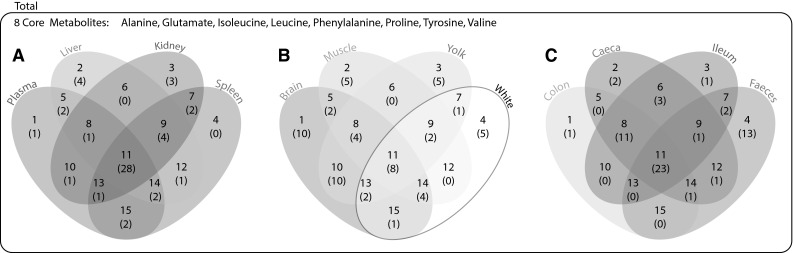
Venn diagram representing metabolic similarities between the 12 studied chicken matrixes. **a** chicken general metabolism: plasma, Liver, Kidney, Spleen. **b** Muscle, egg yolk, egg white and brain cortex. **c** Digestive system: Colon, Caecum, Ileum, Faeces. Each umber represents a zone of intersection that refer to the table presented in Supplementary material 3, the numbers in brackets indicate the number of metabolites shared in the specified zone (between two and four matrixes), details of the metabolites are displayed in Supplementary material 3

Representative ^1^H-NMR spectra of the twelve biological matrices investigated in this study are presented in Figs. 1, 2, 3 and 4 to offer an overview of the chicken metabolome. Organs and biofluids related to: the general metabolism (liver, kidney, plasma and spleen Fig. 1), product destined to consumption (egg yolk and white and muscle Fig. 2), the frontal cortex (Fig. 2) and the lower digestive tract (colon, caecum ileum and faeces Fig. 3). The numerical key for annotation is presented in Table 1 and complementary information provided by 2D spectroscopy for peak assignment is given in Supplementary material 1 and 2.

**Table 1:** ^1^H assignment for identified metabolites and tissue/biofluid. Legend: L, liver; K, kidney; S, spleen; B, cortex; M, pectoral muscle; Ce, caecum; Co, colon; I, ileum; F, faecal water; P, plasma; W, egg white; Y, egg yolk

	Metabolite	Assignement	Matrix
1	2-Hydroxybutyrate	CH_3_ 0.90 t, CH_2_ 1.70 m, CH 4.0 dd	F
2	3-Hydroxybutyrate	CH_3_ 1,19 d, 1/2CH_2_ 2.30 dd, 1/2CH_2_ 2.39 dd, CH 4.14 m	L
3	3-Hydroxyisobutyrate	CH_3_ 1.05 d, CH 2.48 m, 1/2CH_2_ 3.53 dd, 1/2CH_2_ 3.70 dd	F
4	3-Hydroxyphenylacetate	CH_2_COOH 3.47 s, C4H 6.78 m, C6H 6.80 m, C2H 6.85 m, C3H 7.24 t	Co
5	4-Aminobutyrate	βCH_2_ 1.88 m, αCH_2_ 2.29 t, γCH_2_ 3.01 t	B
6	Acetate	CH_3_ 1.92 s	L, K, S, B, M, Ce, Co, I,F, P, W
7	Alanine	βCH_3_ 1.46 d, αCH 3.78 q	L, K, S, B, M, Ce, Co, I,F, P, Y, W
8	β-Alanine	CH_2_COOH 2.56 t, N–CH_2_ 3.19 t	L, K, S, M, Ce, I, P
9	AMP	P–CH_2_ 4.01 m, C1H 4.36 m, C2H 4.50 q, C3H 4.79 t, C4H 6.12 d, C8H 8.25 s, C5H 8.58 s	M
10	Anserine	βCH_2_ 2.68 m, 1/2δCH_2_ 3.03 dd, 1/2δCH_2_ 3.21 dd, αCH_2_ 3.22 m, CH_3_ 3.76 s, γCH_2_ 4.48 m, CH 7.07 s, N–CH 8.20 s	M
11	Arginine	γCH_2_ 1.66 m, βCH_2_ 1.91 m, δCH_2_ 3.27 t, αCH 3.77 t	L, S, P, Y, W
12	Ascorbate	CH_2_ 3.73 ddd, CH 4.01 d, C5 4.51 d	S, B, P
13	Asparagine	1/2βCH_2_ 2.86 dd, 1/2βCH_2_ 2.96 dd, αCH 4.00 dd	L, S, B, Ce, I, Y
14	Aspartate	1/2βCH_2_ 2.68 dd, 1/2βCH_2_ 2.82 dd, αCH 3.91 dd	L, S, Ce, Co, I, F, P, Y
15	Betaine	N–(CH_3_)3 3.37 s, CH_2_ 3.93 s	L, K, S, B, M, Ce, Co, I, F, P, Y
16	Butyrate	CH_3_ 0.88 t, βCH_2_ 1.55 m, αCH_2_ 2.15 t	Ce, Co, I, F
17	Carnitine	αCH_2_ 2.43 m, N–(CH_3_)_3_ 3.21 s, γCH_2_ 3.42 m, βCH 4.56 m	B
18	Carnosine	βCH_2_ 2.67 m, 1/2δCH_2_ 3.03 dd, 1/2δCH_2_ 3.16 dd, αCH_2_ 3.22 m, γCH_2_ 4.46 m, CH 7.08 s, N–CH s	B, M
19	Choline	N–(CH_3_)_3_ 3.22 s, βCH_2_ 3.53 dd, αCH_2_ 4.06 t	L, K, S, B, Ce, Co, I, F, P, Y, W
20	Citrate	1/2γCH_2_ 2.55 d, 1/2γCH_2_ 2.70 d	K, B, I, F, Y
21	Creatine	N–CH_3_ 3.03 s, N–CH_2_ 3.94 s	L, K, S, B, M, Ce, Co, I,F, P, W
22	Creatinine	N–CH_3_ 3.05 s, N–CH_2_ 4.06 s	K, Ce, Co, I, F, P
23	Cysteine	βCH_2_ 3.03 dd, αCH_2_ 3.97 t	S, Ce, Co, I, P
24	Dimethylamine	CH_3_ 2.72 s	F
25	Ethanolamine	CH_2_NH_2_ 3.13 t, CH_2_COH 3.83 t	B, I
26	Formate	HCOOH 8.46 s	L, K, S, B, Ce, Co, I, F, P, W
26	Fumarate	HCOOH 6.51 s	K, S, B, M, Ce, Co, I, P, Y
27	α-Galactose	C6H 3.74 m, C2H 3.80 m, C3H 3.84 m, C4H 3.98 m, C5H 4.07 m, C1H 5.26 d	F, Y
28	β-Galactose	C2H 3.48 m, C3H 3.63 m, C5H 3.69 m, C6H2 3.74 m, C4H 3.92 m, C1H 4.57 d	F, Y
29	α-Glucose	C4H 3.42 m, C2H 3.54 m, CH3 3.72 m, 1/2C6H2 3.73 m, 1/2C6H2 3.77 m, C5H 3.87 m, C1H 5.23 d	L, K, S, M, F, P, Y, W
30	β-Glucose	C2H 3.25 m, C4H 3.49 m, C5H 3.49 m, C3H 3.50 m, 1/2C6H2 3.88 m, 1/2C6H2 3.91 m, C1H 4.66 d	L, K, S, M, F, P, Y, W
31	Glutamate	βCH_2_ 2.02 m, γCH_2_ 2.34 m, αCH 3.76 dd	L, K, S, B, M, Ce, Co, I,F, P, Y, W
32	Glutamine	βCH_2_ 2.15 m, γCH_2_ 2.44 m, αCH 3.77 t	L, K, S, B, M, Ce, Co, I,F, P, Y
33	Glutarate	CH_2_ 1.78 m, 2HCOOH 2.17 t	B
34	Glutathione	CH_2_ 2.17 m, CH_2_ 2.53 m, S–CH_2_ 2.95 dd, N–CH 3.83 m, CH 4.56 q	L
35	Glycerol	1/2CH_2_ 3.58 m, 1/2CH_2_ 3.62 m, CH 3.77 t	L, K, S, B, M, Ce, P, W
36	Glycerophosphocholine	N–(CH_3_)_3_ 3.22 s, NCH_2_ 3.68 m, OCH_2_ 4.32 m	L, K
37	Glycine	αCH_2_ 3.55 s	L, K, S, B, M, Ce, Co, I,F, P, Y
38	Glycogen	C2H 3.63 dd, C4H 3.66 dd, C5H 3.83 q, C6H 3.87 d, C3H 3.98 d, C1H 5.41 m	L
39	Histidine	1/2CH_2_ 3.16 dd, 1/2CH_2_ 3.23 dd, CH 3.98 dd, CH 7.09 s, CH 7.90 s	L, K, S, B, Ce, Co, I, P, Y
40	Hypoxanthine	CH 8.18 s, CH 8.21 s	L, K, S, B, Ce, Co, I, P
41	Inosine	1/2CH_2_ 3.83 dd, 1/2CH_2_ 3.91 dd, C1H 4.27 dd, C2H 4.43 dd, C3H 4.76 t, C4H 6.09 d, NH–CH 8.23 s, N–CH 8.34 s	M, Ce, Co, I
42	Isobutyrate	(CH_3_)_2_ 1.05 d, CH 2.38 m	Ce
43	Isoleucine	γCH_3_ 0.94 t, δCH_3_ 1.02 d, 1/2γCH_2_ 1.26 m, 1/2γCH_2_ 1.47 ddd, βCH 2.01 m, αCH 3.65 d	L, K, S, B, M, Ce, Co, I,F, P, Y, W
44	Lactate	βCH_3_ 1.33 d, αCH 4.12 q	L, K, S, B, Ce, Co, I, F, P, W
45	Leucine	δCH_3_ 0.93 d, βCH_2_ 0.94 d, γCH 1.71 m, αCH 3.73 m	L, K, S, B, Ce, Co, I, F, P, Y, W
46	Lipoproteins (HDL)	C***H***_3_(CH_2_)_n_ 0.84 t, (C***H***_2_)n 1.25 m, C***H***_2_–C=C 2.04 m, C***H***_2_–C–O 2.24 m,=CH–C***H***_2_–CH=2.75 m, CH=CHC***H***_2_ 5.32 m	L,B, F, P, Y
47	Lipoproteins (VLDL)	C***H***_3_CH_2_CH_2_C=0.87 t, C***H***_2_CH_2_CH_2_CO 1.29 m, C***H***_2_CH_2_O 1.57 m, C***H***_2_–C=C 2.04 m, C***H***_2_–C–O 2.24 m,=CH–C***H***_2_–CH=2.75 m, CH=CHC***H***_2_ 5.32 m	L, B, F, P, Y
48	Lysine	γCH_2_ 1.46 m, δCH_2_ 1.71 m, βCH_2_ 1.84 m, εCH_2_ 3.01 t	L, K, S, B, I, F, Y
49	Malate	1/2HCOOH 2.38 dd, 1/2HCOOH 2.66 dd, H–CH 4.30 dd	P
50	α-Mannose	C5H 3.37 m, C4H 3.56 m, C3H 3.65 m, C6H 3.73 m, C2H 3.92 m, C1H 5.17 d	W
51	β-Mannose	C4H 3.65 m, C5H 3.80 m, C3H 3.84, C6H 3.88, C2H 3.92 m, C1H 4.89 d	W
52	Methionine	δCH_3_ 2.13 s, βCH 2.14 m, γCH_2_ 2.60 t, αCH 3.78 t	L, K, S, B, Ce, Co, I, F, P, Y
53	Methylamine	CH_3_ 3.29 s	F
54	*myo*-Inositol	C5H 3.29 t, C1H C3H 3.53 dd, C4H C5H 3.63 t, C2H 4.06 t	L, K, S, B, Ce, Co, I, P, Y, W
55	*N*-Acetylglucosamine	CH3 1.98 s, C3H 3.44&3.76 t, C5H 3.45&3.84 m, C4H 3.48&3.53 t, C2H 3.66&3.86 m, C6H 3.77 m & 3.87 dd, C1H β 4.71 α 5.19 d, NH 8.10 d	F
56	*N*-acetyltyrosine	CH_3_ 1.92 s, 1/2βCH_2_ 2.83 dd, 1/2βCH_2_ 3.08 dd, αCH 4.37 m, C3H C5H 6.84 m, C2H C4H 7.14 m, NH 7.75 d	F
57	Nicotinurate	CH_2_ 3.99 s, H5 7.60 dd, H4 8.25 d, H6 8.71 d, H2 8.94 s	L, K, S, B, M, Ce, Co, I
58	*O*-Phosphocholine	N-(CH_3_)_3_ 3.21 s, CH_2_ 3.58 m, O–CH_2_ 4.16 m	L, K, S, B, Ce, Co, I, Y
59	Ornithine	1/2γCH_2_ 1.72 m, 1/2γCH_2_ 1.82 m, βCH_2_ 1.93 m, δCH_2_ 3.04 t, αCH 3.77 t	K, Y
60	Phenylalanine	1/2βCH_2_ 3.12 dd, 1/2βCH_2_ 3.26 dd, C3H C5H 7.33 m, C4H 7.35 m, C3H C6H 7.40 m	L, K, S, B, Ce, Co, I, F, P, Y, W
61	Proline	γCH_2_ 2.03 m, 1/2βCH_2_ 2.03 m, 1/2βCH_2_ 3.35 m, 1/2δCH_2_ 3.38 m, 1/2δCH_2_ 3.41 m, αCH 4.41 dd	L, K, S, B, Ce, Co, I, F, P, Y, W
62	Propionate	CH_3_ 1.04 t, CH_2_ 2.17 q	Ce, Co, F
63	Serine	αCH 3.85 dd, 1/2βCH_2_ 3.95 dd, 1/2βCH_2_ 3.95 dd	K, S, B, Ce, I, Y
64	*scyllo*-inositol	CH 3.35 s	K
65	Succinate	CH_2_ 2.04 s	L, K, S, M, Ce, Co, I, F, P
66	Taurine	N–CH_2_ 3.26 t, S–CH_2_ 3.43 t	L, K, S, B, Ce, Co, I, P
67	Threonine	γCH_3_ 1.32 d, αCH 3.60 d, βCH 4.25 m	L, K, S, B, Ce, I F, P, Y
68	Trigonelline	CH_3_ 4.43 s, C4H 8.07 m, C3H C5H 8.91 m, C1H 9.11 s	F
69	Trimethylamine *N*-oxide	N–(CH_3_)_3_ 3.27 s	L, K, B, Ce, Co, I, F, P
70	Tryptophan	1/2βCH_2_ 3.31 dd, 1/2βCH_2_ 3.49 dd, αCH 4.06 dd, C5H 7.21 t, C6H 7.29 t, C1H 7.33 s, C3H 7.55 d, C4H 7.74 d	L, K, S, Ce, Co, I, F, Y
71	Tyrosine	1/2CH_2_ 3.04 dd, 1/2CH_2_ 3.18 dd, N–CH 3.94 dd, C3H C5H 6.89 m, C2H C6H 7.18 m	L, K, S, B, Ce, Co, I, F, P, Y, W
72	UDP-glucose	C4H 3.47 t, C2H 3.54 m, C3H 3.77 t, 1/2C6H 3.77 dd 1/2C6H 3.85 dd, C5H 3.88 m, 1/2CH_2_ 4.19 m, 1/2CH_2_ 4.24 m, O–CH 4.28 m, C’3H 4.36 dd, C’2H 4.37 dd, C1H 5.97 d, O–CH–N 5.97 d, N–CH 7.94 d	W
73	UDP-*N*-acetyl glucose	CH_3_ 2.07 s, C4H 3.55 t, C3H 3.80 t, 1/2C6H 3.81 dd, 1/2C6H 3.86 dd, C5H 3.91 m, C2H 3.98 m, 1/2CH_2_ 4.18 m, 1/2CH_2_ 4.23 m, O–CH 4.28 m, C’3H 4.35 dd, C’2H 4.36 dd, C1H 5.51 dd, CH 5.95 d, O–CH–N 5.97 d, N–CH 7.94 d, NH 8.35 d	W
74	Uracil	C5H 5.80 d, C6H 7.54 d	L, K, S, B, Ce, Co, P
75	Uridine	1/2CH_2_ 3.81 dd, 1/2CH_2_ 3.92 dd, C4H 4.12 dt, C3H 4.24 dd, C2H 4.36 dd, C1H 5.88 d, C5H 5.92 m, C6H 7.88 d	W, S
76	Valerate	CH_3_ 0.88 t, γCH_2_ 1.29 m, βCH_2_ 1.51 m, αCH_2_ 2.17 t	Ce, F
77	Valine	γCH_3_ 0.98 d, γ’CH_3_ 1.04 d, βCH 2.27 m, αCH 3.62 d	L, K, S, B, Ce, Co, I, F, P, Y, W
78	Xanthine	CH 7.92 s	K, S, B, Ce, Co, I

### Matrix characterization

The hepatic metabolic profile (Fig. 1a) was characterised by high levels of betaine, lactate and glucose. This was the only biological matrix where it was possible to detect glutathione (in its oxidised form since the total pool of glutathione becomes oxidised during tissue extraction), in very small quantities, in contrast to what is commonly found in mammalian hepatic metabolic profiles (Martin et al. 2007; Waters et al. 2002; Duarte et al. 2005; Claus et al. 2008).

Similarly, kidney metabolic profiles (Fig. 1b) were rich in lactate, which is consistent with the important role of the kidney in energy metabolism. In addition, betaine and creatine were found in very high concentrations. Betaine is an important osmolyte in the kidney and its concentration generally increases in case of water privation such as diarrhoea resulting from infection. In birds the most important kidney osmolytes are *myo*-inositol, betaine, glycerophosphorylcholine, and taurine(Lien et al. 1993) that were all detected using ^1^H-NMR.

The metabolic profile of the spleen was characterized by high levels of betaine, *myo*-inositol and phosphocholine (Fig. 1c). This was one of the few matrices that did not possess any unique metabolic feature, as all the metabolites detectable by NMR spectrometry were shared with liver, kidney and plasma. This similarity may be explained by the high vascularization of this tissue. In particular, it shared with plasma high lactate and betaine levels. Unique to plasma metabolic fingerprints were large resonances from lipoproteins, mainly HDL and VLDL (Fig. 1d). It was also possible to see high lactate, glucose and betaine levels. Its metabolic profile was similar to liver, kidney and spleen, but it was the only matrix where it was possible to identify malate, derived from the metabolism of the citric acid cycle.

The pectoral muscle presented the most distinctive metabolic features in respect to the other tissue type samples, with only twenty-three identifiable metabolites (Fig. 2a). Three metabolites were in noticeably high concentration: anserine, creatine and lactate. We only detected AMP in muscle. Due to its pKa close to 7 anserine is a very good buffer that maintain muscle pH neutrality (Boldyrev et al. 2013). The ability of anserine to maintain a certain pH in the muscle is known to increase the rate of glycolysis (Davey et al. 1960). It is also a well-known antioxidant (Kohen et al. 1988), playing an important role during muscle contraction.

The metabolic profile of egg white had high glucose content and presented only twenty-three detectable metabolites (Fig. 2b). This was not surprising knowing that egg white is relatively poor in micronutrient and is mainly constituted of water (88 %), protein (10 %) and less that 1 % of carbohydrates (Reserves 2007). Egg nutritive values for embryo development are mainly attributed to these proteins (Reserves 2007). It was also the only matrix where we could detect glucose derived molecules, such as uridine diphosphate glucose (UDPG) involved in embryo retina development (Dreyfus et al. 1975) and UDP-*N*-acetyl glucosamine (UDP-GlcNAC) as previously described by Donovan et al. (Donovan et al. 1967) that can be associated with muscle expansion (Ullrich et al. 1981). UDPG is involved in polysaccharide synthesis and UDP-GlcNAC is related to glycosaminoglycan, proteoglycan and glycolipid anabolism but nothing specific to its role in eggs could be found in the published literature.

In contrast, yolk polar phase metabolic profile featured high levels of amino acids and carbohydrates such as glucose and galactose (Fig. 2c). All amino acids essential for protein synthesis but cysteine (that can be generated from methionine or serine) were detectable in the yolk as well as residual lipids that constitute 66 % of yolk dry matter (Reserves 2007). No particularly distinctive metabolites were observed in the yolk.

The metabolic profile of the cortex presented a high content in *myo*-inositol, creatine, glutamate, taurine and 4-aminobutyrate (GABA) (Fig. 2d). Carnosine was also detected, which is a known brain antioxidant (Kohen et al. 1988). Surprisingly in contrast with muscle, it was not possible to detect anserine, which has been reported to be present in birds central nervous system(Biffo et al. 1990).

The metabolic profiles of gastrointestinal segments were characterised by the presence of amino acids and short chain fatty acids (SCFAs) (Fig. 3). A distinctive feature of the ileum was the presence of glucose (Fig. 3c). Furthermore, the aromatic region was richer in phenylalanine and tyrosine than colon and caecum. The ileum did not present any unique metabolic feature. The metabolic profile of the caecum contained high levels of short chain fatty acids and amino acids (Fig. 3b). It was also possible to detect isobutyrate a product of amino acid degradation by gut bacteria. A very high level of *O*-phosphocholine, which has been associated with an immunologic response to bacterial infections (Wiens et al. 2003), was observed in this tissue. The metabolic profile of the colon (Fig. 3a) was high in short chain fatty acids (acetate, propionate and butyrate) and amino acids (alanine, aspartate, glutamate, glutamine, glycine, histidine, isoleucine, leucine, methionine, phenylalanine, proline, tryptophan, tyrosine and valine). It was the only tissue where we detected 3-hydroxyphenylacetate. Unlike previously published results for rodents (Claus et al. 2008), glucose resonances were not visible in the colon, despite its presence in faeces. Colon was the digestive system related matrix presenting the poorest metabolic diversity with thirty-six detectable metabolites. Finally, in birds, faeces also contain urine since the digestive and urinary systems share the same portal (the cloaca). Therefore, it was not surprising to observe forty-three metabolites, of which only ten of them pertained exclusively to faeces: 2-hydroxybutyrate, 3-hydroxyisobutyrate, arabinose, benzoate, dimethylamine, methylamine, *N*-acteylglucosamine, *N*-acetyltyrosine and trigonelline (Fig. 3d).

### Matrix cross comparison

Cross tissues comparison of detectable metabolites was performed using a Venn diagram (Fig. 4 and Supplementary material 3) and revealed the high metabolic variability existing between the twelve biological matrices investigated in this study. Only eight core metabolites were found out of a total of seventy-eight detected molecules. Detected core metabolites were all amino acids: alanine, glutamate, isoleucine, leucine, phenylalanine, proline, tyrosine and valine and can be considered ubiquitous stable metabolites. Matrices related to general metabolic processes (liver, kidney, spleen and plasma) shared twenty-eight metabolites related to energy and protein metabolism. Biological matrices related to the digestive system (colon, caecum, ileum and faeces) shared 23 core metabolites associated with microbial activity, energy metabolism and protein degradation.

**Fig. 5:**
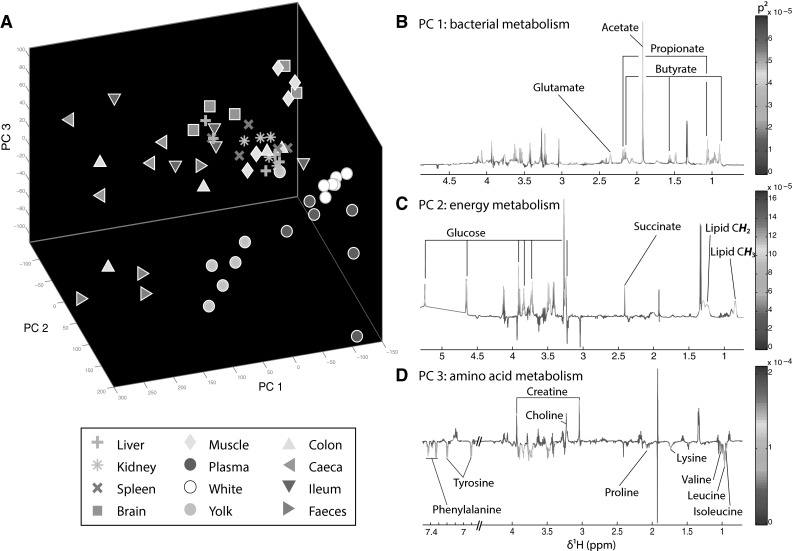
Metabolic variability between the twelve described chicken matrices. **a** 3-Dimentional PCA score plot derived from the ^1^H-NMR spectra of liver, kidney, spleen, brain, muscle, plasma, white, yolk, colon, caecum, ileum and faeces of six animals. **b** PCA loadings representing the metabolic variations on PC1. **c** PCA loadings representing the metabolic variations on PC2. **d** PCA loadings representing the metabolic variations on PC3

The largest source of metabolic variation between the twelve biological matrices was visualised using PCA (Fig. 5a). The scores of liver, kidney and spleen samples were clustered together on the three first principal components representing 77 % of the total variance (PC1, PC2 and PC3, Fig. 5a). Surprisingly, this was also observed for muscle and brain cortex tissues. Metabolic profiles of samples derived from the digestive system were also grouped together but presented the highest variability between samples of the same matrix. These were the samples driving separation on the first component, which was associated with increased levels in short chain fatty acids produced by gut microbial activity. Finally, plasma, egg yolk and egg white were clustered together on PC2 due to their high glucose content. Yolk and plasma metabolic profiles also clustered together because they shared high lipid levels. Interestingly, egg-derived samples were the most metabolically homogenous, with the least inter-individual variability indicating that their metabolism is tightly regulated.

The metabolic profiles of colon, caecum, ileum and faecal water shared many similar metabolic patterns. 23 shared metabolites were related to microbial catabolism of polysaccharides (acetate, butyrate) and protein degradation (amino acids). Propionate, another important product of polysaccharide fermentation was not found in the ileum but was observed in all other digestive matrices, indicating that propionate fermentation does not occur in this part of the digestive system. It was not possible to separate caecum and colon metabolic profiles using pairwise comparison such as orthogonal projection to latent structure discriminant analysis (O-PLS DA) due to their high metabolic similarity. However, it was possible to distinguish the ileum from colon and caecum based on lower SCFAs concentration, which suggests that gut microbiota (GM) at this level of the gastro intestinal (GI) tract is less active (Fig. 5b). The same was observed in mice where more SCFAs were found in the lower part of the GI tract due to high microbial colonization (Martin et al. 2009a, b). This metabolic characteristic clearly separated the cluster of GI samples from the other matrices on the PCA plot. Faecal water was the biofluid presenting the highest quantity of identifiable metabolites, of which ten were uniquely found in this matrix probably as a result of the complexity of the food provided (see Supplementary material 4 and Fig. 5a and b) and high microbial activity. These ten metabolites were mostly SCFAs, likely derived from gut microbiota activity as well as methyl donors including methylamines. The high similarity level existing between GI tract metabolic profiles and faecal waters indicates a great level of exchange between the GI lumen and the enterocytes. Birds were fed with un-medicated layer pellets (Dodson and Horrell) that mainly contain wheat rich in complex carbohydrate, vegetable oil and soya as a protein source (for more information see Supplementary material 4).

Highly metabolically active tissues, liver, kidney and spleen, appear to be very similar although they serve different purposes (i.e. spleen is more involved in immune control) as presented on the PCA plot. However, due to the high number of studied matrices and their high variability, this model lacks of sensitivity to separate the three tissues which present a high level of metabolic similarity, both qualitatively and quantitatively. Nevertheless, they also present distinct features such as glucose and creatine levels that were detectable using pairwise comparisons and PCA (Supplementary material 4).

Egg metabolic profiles were dominated by energy metabolites (saccharides) and amino acids for both yolk and white matrices. Yolk was also extremely rich in cholesterol and lipids, which are essential to cell membrane formation and are also sources of energy (Yeagle 1989; Spector and Yorek 1985). These results confirm the high nutritive value of chicken eggs due to their initial purpose to support fetal development.

The metabolic profile of muscle has only been described in mice for cardiac muscle (Griffin et al. 2001), which in its structure and function is different to striated skeletal muscle. Despite their differences, both muscle metabolic profiles appear to be characterized by lactate, which is the main product of glucose anaerobic fermentation by muscle during exercise (Brooks 1986). Creatine was also found in high concentration, which is consistent with its important role as a phosphate donor to quickly regenerate ATP during muscular contraction (Bessman and Geiger 1981; Casey et al. 1990). Finally taurine, also involved in contractility, was highly concentrated (Pierno et al. 1998).

In comparison to previously described metabolic profiles of mammals from mice, pigs and humans, these profiles show high qualitative but not necessarily quantitative similarities for liver, kidney, ileum, colon and plasma. This shows that despite the level of genetic and evolutionary differences existing between birds and mammals, their core metabolic functions remain very similar. The main difference previously mentioned between chicken and mammalian metabolic profiles were observed in the liver where we observed that glutathione levels were noticeably lower in birds. Glutathione is involved in cell protection due to its antioxidant properties (Meister 1983). This difference had been already reported in quail (Gregus et al. 1983), suggesting a major shift in hepatic detoxification mechanisms between mammals and birds. Indeed, several publications have reported a higher susceptibility of birds to toxic substances and a higher bioaccumulation in comparison to mammals (Walker 1983) consistent with a modification of detoxification metabolism during evolution.

## Conclusion

This study presents a large overview of chicken metabolic profiles in various tissues and biofluids that could be used as a database for future NMR-based metabonomic analyses in avian studies. Future works focussing on the metabolic impact of GI infection and treatment on host metabolism and on the influence of diet and growth condition would be useful to assess product quality (i.e. meat and egg). These metabolic data integrated with other ‘omics’ approaches will contribute to the understanding of host response to environmental changes, infection and treatment that should lead to improved animal welfare.

## Electronic supplementary material

Below is the link to the electronic supplementary material.

Supplementary material 1 (EPS 1571 kb)

Supplementary material 2 (EPS 1600 kb)

Supplementary material 3 (XLSX 25 kb)

Supplementary material 4 (EPS 15362 kb)
